# Supplementary material for: The best features of diamond nanothread for nanofibre applications
Source: Nat Commun. 2017 Mar 17;8:14863. doi: 10.1038/ncomms14863 (PMC5357841; doi:10.1038/ncomms14863)
Supplement: Supplementary Information — Supplementary Figures, Supplementary Tables and Supplementary References [file ncomms14863-s1.pdf]

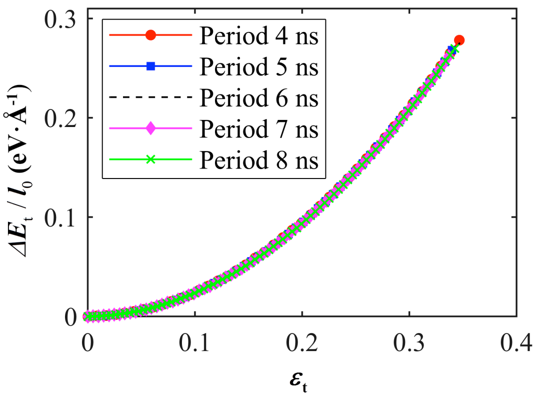

Supplementary Figure 1 | Twist energy per length as a function of the dimensionless twist rate for DNT bundle. Five loading rates with a period of 4, 5, 6, 7, and 8 ns have been examined. The energy profiles are highly overlapped with each other, with an averaged elastic limit about 0.34 ± 0.4%.

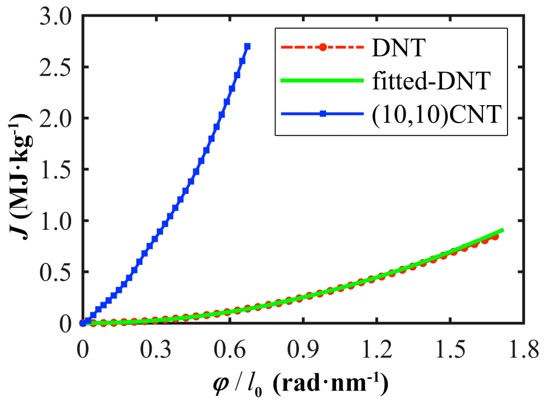

Supplementary Figure 2 | Gravimetric deformation energy density *J* as a function of the twist rate $\varepsilon_{t0} = \varphi / l_0$ for individual DNT and (10,10) CNT. Green solid line is the fitting curve, which is fitted with the twist rate within 0.6 rad·nm^{-1} and then extended to higher twist rate. For (10,10) CNT, we adopted the torsional rigidity from *ab initio* density functional theory (DFT) calculations,[1] which is about 18689 eV·Å (by considering the radius of 6.78 Å). In comparison, a very small torsional rigidity about 20 eV·Å is obtained for the DNT by fitting the energy density curve according to the energy relationship with the twist angle ($\Delta E_t = GI_p\varphi^2 / 2l_0$). Following the solid cylinder approximation for the DNT, its polar moment of inertia can be calculated as $\pi d^4 / 32$ (here d is the diameter of the DNT), which is about 28.33 Å^4. In the meanwhile, the (10,10) CNT can be considered as a circular hollow shaft with a thickness (t) of 3.35 Å (as commonly used in previous studies). In other words, its polar moment of inertia can be calculated from $\pi[(D+t)^4 - (D-t)^4]/32$ (here D is the diameter of the CNT), which yields to 7080.7 Å^4. That is, the shear modulus for the DNT and (10,10) CNT approximate to 114 GPa and 423 GPa, respectively.

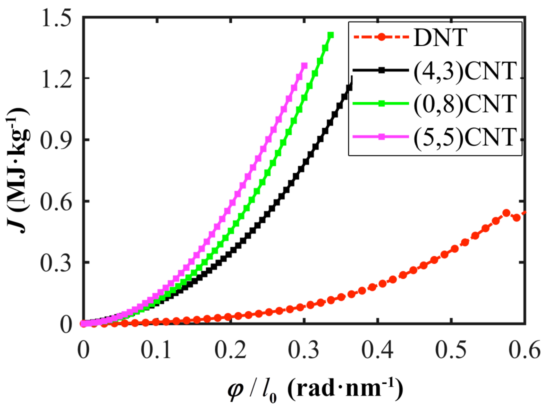

Supplementary Figure 3 | Gravimetric deformation energy density *J* as a function of the twist rate $\varepsilon_{t0} = \varphi / l_0$ for individual DNT and different thinner CNTs. Examined CNTs include (4,3) CNT, (0,8) CNT, and (5,5) CNT.

Supplementary Table 1 | Torsional properties of bundles constructed from DNT and CNTs.

Bundle type	DNT	(4,3)CNT	(0,8)CNT	(5,5)CNT	(10,10)CNT
Torsional elastic limit (rad·nm^{-1})	0.57	0.37	0.34	0.30	0.16
Torsional rigidity (eV·Å)	325	8579	14779	20523	$1.65 * 10^4$

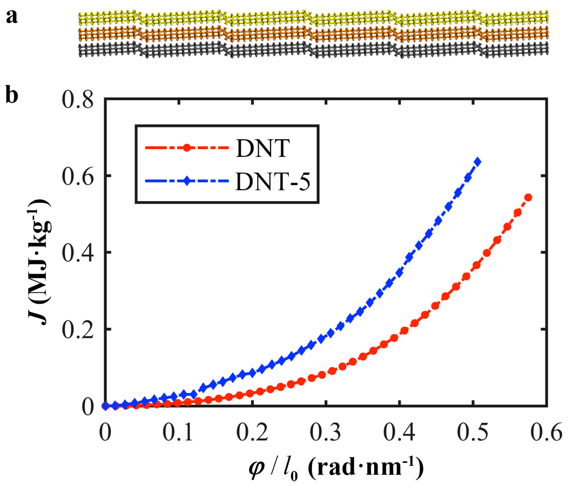

Supplementary Figure 4 | Twist deformation for bundle constructed from DNT with less SWD. (a) The bundle made from DNT-5 (with only 5 SWDs). (b) Gravimetric deformation energy density J as a function of the twist rate $\varepsilon_{t0} = \varphi / l_0$ for DNT and DNT-5 bundles.

Supplementary Table 2 | The six adjacent contacts in the armchair (m,n) CNT lattice as observed from the tests of an infinite triangular lattice of CNTs with sixfold symmetry. SP^1 is the transition status between two AA stacking configurations, and SP^2 is the transition status between two AB stacking configurations.

m/*n*	6	7	8	9	10	11	12
AA	-	-	2	-	2	-	-
AB	-	-	4	6	4	-	6
SP^1	-	2	-	-	-	2	-
SP^2	6	4	-	-	-	4	-

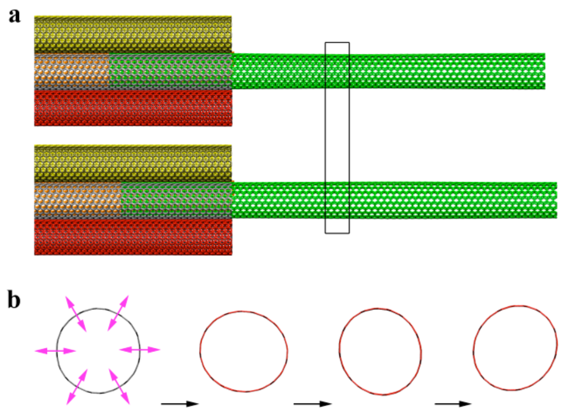

Supplementary Figure 5 | Radial breathing mode of CNT. (a) The atomic configurations of the DNT bundle during pull-out. (b) Morphology of the cross-section of the core CNT at different displacement, showing the occurrence of RBM (which is seen in the potion being that is pulled out from the bundle structure). For a bundle structure, the RBM of the core CNT will alter the intertubule pressure, and thus affect the interface friction.[2] Previous studies show that the frequency of RBM ω_{RBM} is dependent on the diameter of CNT for isolated CNTs, $\omega_{\mathrm{RBM}} = C_1 / D^k + C_2(D)$.[3] Here C_1 and C_2 are constant, and k is an exponent. For (10,10) CNT,[4] the RBM frequency is about 175 cm^{-1}, which is much higher comparing with the transition period of the commensurate interface. According to the atomic configurations, the RBM of the core CNT is suppressed when it is embedded in the fixed surrounding CNTs, and becomes significant for the portion being pulled out. Since the RBM is interrelated with the interface vdW interaction,[2] a simple isolation of its influence on the transferred load is unavailable, which deserves further investigation.

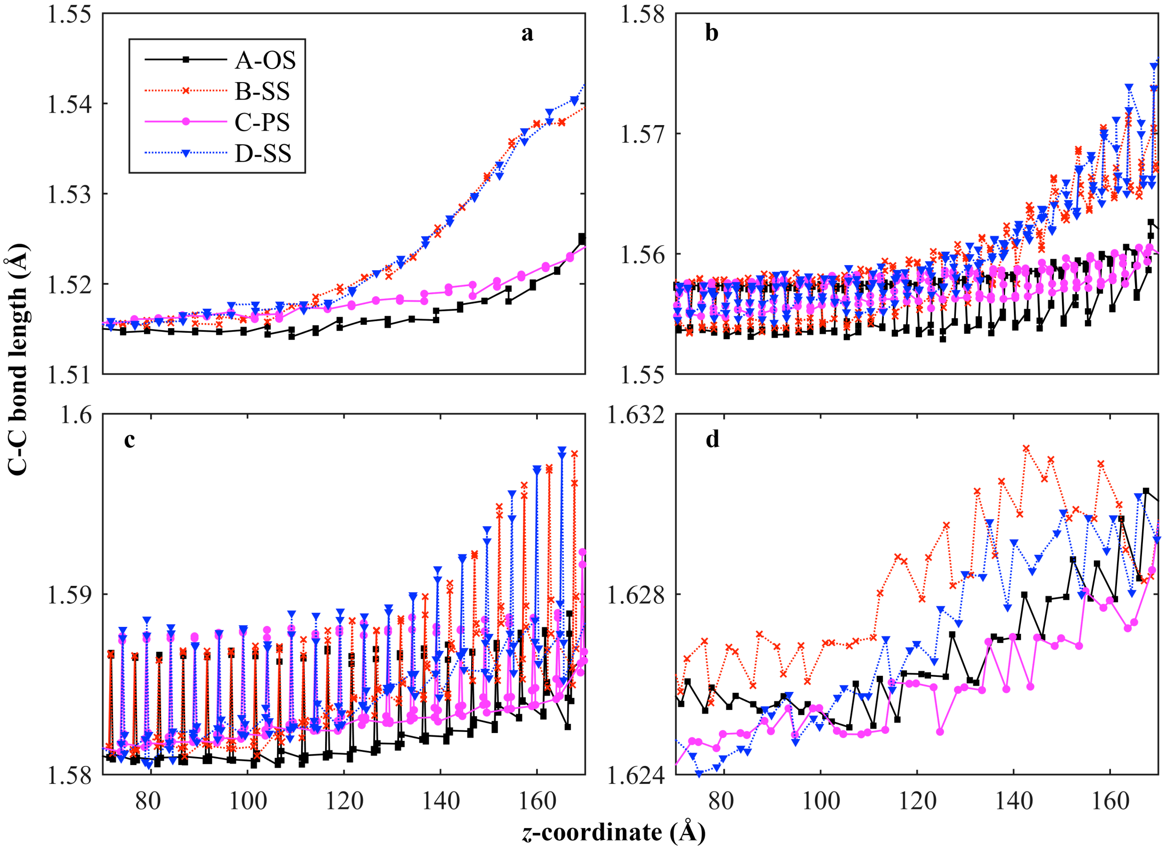

Supplementary Figure 6 | C-C bond length distribution of the core DNT during the stick-slip motion (OS-SS-PS-SS). In the range of: (a) 1.51 ~ 1.55 Å, (b) 1.55 ~ 1.58 Å, (c) 1.58 ~ 1.60 Å, and (d) 1.624 ~ 1.632 Å. In all four ranges, the C-C bond length (near the fixed end of the six surrounding DNTs) in the two strained status (SS) is longer than the energy minimum statuses (OS and PS). This result indicates strong local stretch in the DNT during the stick period.

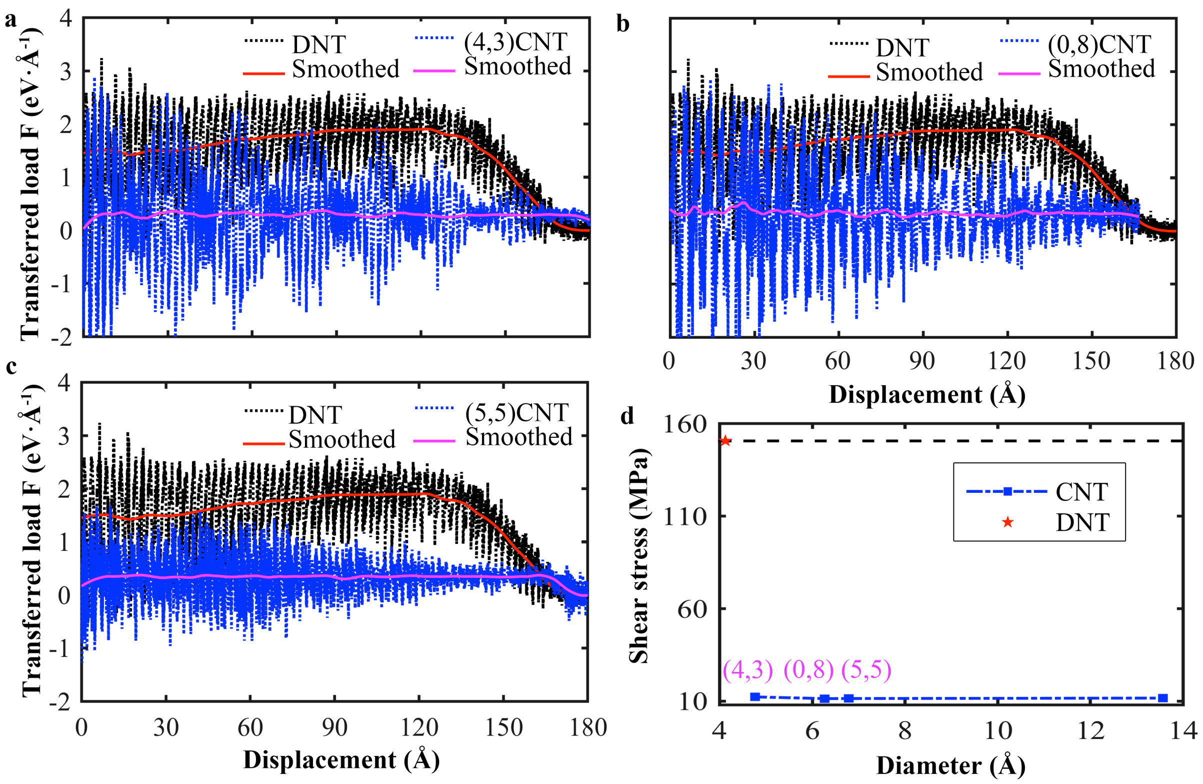

Supplementary Figure 7 | Sliding simulation for different CNT bundles. Comparison of the transferred load *vs* the displacement of the end of the core strand for: (a) DNT and (4,3) CNT bundles; (b) DNT and (0,8) CNT bundles; (c) DNT and (5,5) CNT bundles (solid lines are the corresponding smoothed curves), and (d) The estimated interface shear stress, which fluctuates around 11.5 MPa and decreases gradually with the increasing diameter of the constituent CNT.

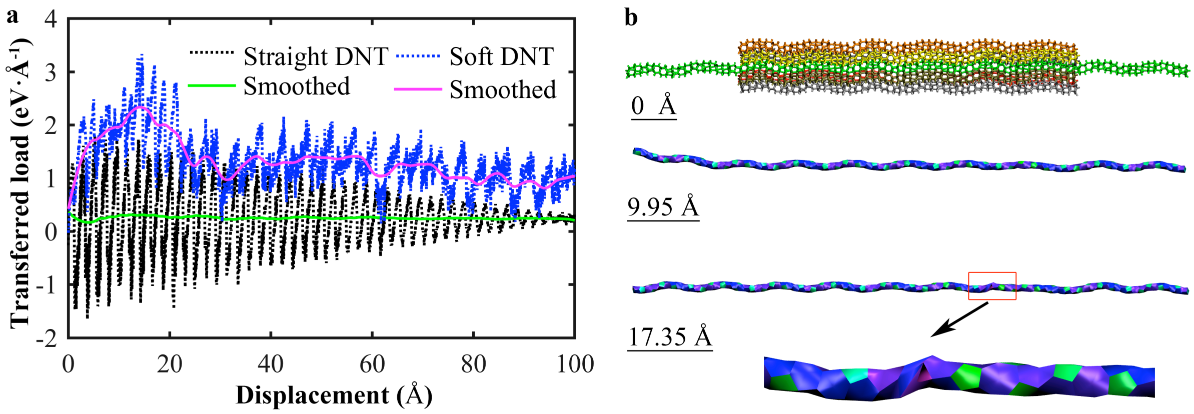

Supplementary Figure 8 | Sliding simulation for different DNT bundles. (a) Comparison of the transferred load *vs* the displacement of the end of the core strand for straight DNT and soft DNT bundles; (b) Atomic configurations of the soft DNT bundle, which shows the bond breaking during sliding process. Only the core DNT is visualized in the middle and bottom images and they are visualized using paperchain method.

Supplementary References

1 Teich, D., Fthenakis, Z. G., Seifert, G. & Tománek, D. Nanomechanical Energy Storage in Twisted Nanotube Ropes. *Phys. Rev. Lett.* **109**, 255501 (2012).

2 Venkateswaran, U. D. *et al.* Probing the single-wall carbon nanotube bundle: Raman scattering under high pressure. *Phys. Rev. B* **59**, 10928-10934 (1999).

3 Machón, M. *et al.* Strength of radial breathing mode in single-walled carbon nanotubes. *Phys. Rev. B* **71**, 035416 (2005).

4 Kürti, J., Kresse, G. & Kuzmany, H. First-principles calculations of the radial breathing mode of single-wall carbon nanotubes. *Phys. Rev. B* **58**, R8869-R8872 (1998).
